# Single nucleotide polymorphisms in the *ETS1* gene are associated with idiopathic inflammatory myopathies in a northern Chinese Han population

**DOI:** 10.1038/s41598-017-13385-1

**Published:** 2017-10-13

**Authors:** Si Chen, Xiaoting Wen, Liubing Li, Jing Li, Yuan Li, Qian Wang, Hui Yuan, Fengchun Zhang, Yongzhe Li

**Affiliations:** 1 Department of Rheumatology and Clinical Immunology, Peking Union Medical College Hospital, Chinese Academy of Medical Sciences & Peking Union Medical College, Key Laboratory of Rheumatology and Clinical Immunology, Ministry of Education, Beijing, China; 2 Department of Clinical Laboratory, Beijing Anzhen Hospital, Capital Medical University, Beijing, China

## Abstract

Single-nucleotide polymorphisms (SNPs) in the *ETS1* gene are associated with several auto-inflammatory diseases. In this study, we determined whether *ETS1* gene polymorphisms confer susceptibility to idiopathic inflammatory myopathies (IIMs) in a northern Chinese Han population. DNA samples were collected from 1017 IIM patients: 363 PM cases and 654 DM cases. The results were compared with those of 1280 healthy controls. Five SNPs in the *ETS1* region (rs7117932, rs6590330, rs4937362, rs10893845 and rs1128334) were assessed and genotyped using the Sequenom platform. Our data indicated that the rs7117932 alleles and genotypes are associated with DM and IIMs (*P*_*c*_ = 6.0 × 10^−3^ and *P*_*c*_ = 0.029; *P*_*c*_ = 0.013 and *P*_*c*_ = 0.019, respectively). We found a significantly greater percentage of DM and IIM patients with an A allele of rs6590330 than that in the control population (*P*_*c*_ = 0.033 and *P*_*c*_ = 0.013). Additionally, the rs6590330 genotype was associated with IIMs (*P*_*c*_ = 0.020). The percentages of rs7117932 and rs6590330 SNPs were significantly greater in DM and IIM patients with interstitial lung disease (ILD) (all *P*_*c*_ < 0.05). This is the first study to reveal that *ETS1* polymorphisms are associated with IIMs alone and IIMs with ILD in a northern Chinese Han population.

## Introduction

Idiopathic inflammatory myopathies (IIMs) are a heterogeneous group of rare systemic diseases that are primarily characterized by the presence of symmetrical, proximal muscle weakness, inflammatory infiltrates in skeletal muscle tissue and elevated levels of skeletal muscle enzymes. Patients may also present with extra-muscular features, including skin rashes, interstitial lung disease (ILD) and malignancy, that are often related to serum antibody status^1^. Polymyositis (PM) and dermatomyositis (DM) are the most common clinical subtypes of IIMs^2^. The etiology of IIMs is poorly understood, and these diseases may be complex multi-factorial genetic diseases caused by immune activation and specific environmental factors in genetically susceptible individuals.

Because IIMs are a group of rare autoimmune diseases, difficulties have been reported in previous genetic association and candidate gene studies. Prior studies have demonstrated a substantial genetic risk of IIMs associated with the major histocompatibility complex (MHC) gene region and, specifically, the 8.1 ancestral haplotype (8.1 AH)^3^. Several recent genome-wide association studies (GWAS) of IIMs have confirmed that the MHC is the major genetic region associated with IIMs and have shown that IIMs share non-MHC genetic regions with other autoimmune diseases^4–6^. The *ETS1* gene is a 63 kb gene consisting of 8 exons located on chromosome 11q24.3. *ETS1* gene variants are associated with susceptibility to various human inflammatory and autoimmune disorders in addition to several obesity-related conditions including rheumatoid arthritis (RA), systemic lupus erythematous (SLE), ankylosing spondylitis (AS), multiple sclerosis, and celiac disease^7–14^. Two extensively studied single-nucleotide polymorphisms (SNPs) (rs7117932 and rs6590330) are correlated with several rheumatic diseases^7–10^. Ets1 is a transcription factor that plays multiple and diverse roles in regulating the differentiation of immune cells and other cell types. For example, Ets1 controls T-cell differentiation and directly regulates the expression of various cytokines and chemokines^15^. Ets1 is also a negative regulator of terminal B-cell differentiation, and Ets1-deficient B cells are present in normal numbers but show a large proportion of IgM-positive plasma cells^16^.

We designed this case-control study to test the hypothesis that variants of the *ETS1* gene might predispose northern Chinese Han patients to IIMs due to the role of this gene in immune functions. Our aim was to uncover new insights into the genetic structure of IIMs and determine the genetic characteristics of different clinical subgroups.

## Materials and Methods

### Samples

This study enrolled 1017 IIM patients and 1280 ethnically matched controls. We obtained written informed consent from all participants. The patients were recruited from two different sources. The study consisted of 569 patients, 184 PM patients and 385 DM patients, enrolled from the Peking Union Medical College Hospital. This study was supported by the Research Special Fund for Public Welfare Industry of Health, and 448 patients, consisting of 179 PM patients and 269 DM patients, were recruited through the cooperation of several centers in northern China. Overlap exists between these samples and previous IIM genetic studies^17–20^. IIM patients were included if they fulfilled the probable or definite Bohan and Peter classification criteria for IIM^21,22^. A total of 1280 ethnically matched healthy controls were recruited from the Peking Union Medical College Hospital during their physical examinations using the following inclusion criteria: 1) no significant history of rheumatologic disease; 2) no family history of rheumatologic diseases; 3) normal biochemical and immunological profiles; and 4) negative serology for anti-Jo-1 and anti-Mi-2 antibodies. The approval for this study was obtained from the Ethics Committee of the Peking Union Medical College Hospital (Beijing, China).

### Selection of SNPs

*ETS1* has several functions in rheumatic immune diseases. Five SNPs (rs7117932, rs6590330, rs4937362, rs10893845 and rs1128334) of this gene that have previously been associated with other autoimmune diseases based on GWAS or candidate gene studies were evaluated in our study. All five SNPs met the Agena design standards and were genotyped using the Agena MassArray iPLEX system (Sequenom, San Diego, CA, USA).

### DNA extraction and SNP genotyping

A 2-mL peripheral blood specimen was collected from all participants, and DNA was extracted according to the manufacturer’s instructions using a genomic DNA kit (Tiangen; Beijing, China). The five SNPs of the *ETS1* gene were genotyped with the MassArray iPLEX system (Sequenom, San Diego, CA, USA) according to the manufacturer’s protocol. Briefly, after multiplex PCR amplifications, the products were used for locus-specific single-base extension reactions. The final products were desalted and transferred to 384-element SpectroCHIP arrays (Sequenom). The alleles were detected using matrix-assisted laser desorption/ionization-time-of-flight mass spectrometry (MALDI-TOF MS). The resultant mass spectrograms and genotype data were analyzed with MassArray Typer 4.0 software. The primers for polymerase chain reaction (PCR) and single-base extension analysis were designed with MassArray Assay Design 4.0 (Sequenom). All methods were performed in accordance with the relevant guidelines and regulations

### Statistical analysis

Each SNP was examined for Hardy-Weinberg equilibrium (HWE) in control population, and any SNPs that deviated significantly from the HWE (*P* < 0.05) were excluded from further analysis. The association analyses between the case and control groups were performed using PLINK v1.07 software (Shaun Purcell; Boston, MA, USA)^23^. The odds ratio (OR) and 95% confidence interval (95% CI) were calculated, and *P*-values < 0.05 were considered statistically significant. The results were corrected for multiple testing using the Bonferroni adjustment. In addition, additive, dominant, and recessive logistic regression genetic models were used to analyze the five SNPs. The sub-phenotype stratification analyses included the association study for *ETS1* polymorphisms and the presence of ILD.

## Results

### Clinical characterization of subjects

The clinical characteristics of the patients and controls are shown in Table 1. The study enrolled 1017 IIM patients (74.3% female): 363 PM patients and 654 DM patients. The mean age of the IIM patients was 46.1 ± 15.2 y. The healthy controls included 1280 subjects (87.1% female; mean age 41.8 ± 12.7 y). The success rate of genotyping for all subjects was greater than 97%, except for rs10893845. All SNPs were in HWE in healthy controls (P_HWE_ > 0.05), except for rs1128334. Therefore, rs10893845 and rs1128334 were excluded from further analysis. The Genetic Power Calculator revealed that our sample size had greater than 80% power (α = 0.05) for detecting an association with a relative risk of 1.2–1.5 for both heterozygotes and homozygotes. The calculations used assumptions of 0.001% PM/DM prevalence in the Chinese population and a risk allele frequency of 0.30 (similar to the allele frequencies of the tested SNPs in an Asian study).

**Table 1:** Clinical data of IIM patients and controls.

Characteristic	Patients	Controls
Number of subjects (DM/PM)	1017(654/363)	1280
Female percentage (%)	74.3	87.1
Average age (mean ± SD)	46.1 ± 15.2	41.8 ± 12.7
DM with ILD, No./total (%)	390/654(59.6)	—
PM with ILD, No./total (%)	195/363(53.7)	—

PM: polymyositis; DM: dermatomyositis; IIM: idiopathic inflammatory myopathies; ILD: interstitial lung disease.

### Genetic analysis

The risk allele and genotype frequencies of the PM and DM patients were compared to those of healthy controls (Table 2). The rs7117932 alleles and genotypes were associated with DM and IIM patients (*P*_*c*_ = 6.0 × 10^−3^, OR = 1.26 and *P*_*c*_ = 0.029; *P*_*c*_ = 0.013, OR = 1.21 and *P*_*c*_ = 0.019, respectively). The percentages of DM and IIM patients with an A allele consisting of rs6590330 were also significantly greater than those in the healthy control group (*P*_*c*_ = 0.033, OR = 1.19 and *P*_*c*_ = 0.013, OR = 1.19). Additionally, the rs6590330 genotype was associated with IIM patients (*P*_*c*_ = 0.020). However, no statistically significant differences were observed in the allele or genotype frequencies of rs4937362 between DM, PM, and IIM patients and the healthy controls (all *P*_*c*_ > 0.05; Table 2). Statistical analyses using logistic regression with genetic additive, dominant, and recessive models showed similar patterns (Table 3). Significant associations were observed for rs7117932 and rs6590330 with DM and IIM patients in the additive and dominant models. The SNP rs6590330 was associated with PM patients only in the dominant model.

**Table 2:** Allele and genotype distribution of the *ETS1* gene markers in IIM patients and controls.

SNPs	Groups	Allele (%)		OR (95%CI)	*P*	*Pc*	Genotype (%)			χ^2^	*P*	*Pc*
rs6590330		A	G				AA	GA	GG			
	DM	490(37.5)	818(62.5)	1.19(1.04–1.38)	0.011	**0.033**	85(13.0)	320(48.9)	249(38.1)	6.80	0.033	0.099
	PM	271(37.3)	455(62.7)	1.19(1.00–1.41)	0.047	0.141	40(11.0)	191(52.6)	132(36.4)	7.12	0.028	0.084
	IIM	761(37.4)	1273(62.6)	1.19(1.06–1.35)	4.2 × 10^−3^	**0.013**	125(12.3)	511(50.2)	381(37.5)	10.0	6.7 × 10^−3^	**0.020**
	Controls	854(33.4)	1706(66.6)				137(10.7)	580(45.3)	563(44.0)			
rs7117932		T	C				TT	TC	CC			
	DM	388(29.7)	920(70.3)	1.26(1.08–1.46)	2.0 × 10^−3^	6.0 × 10^−3^	56(8.6)	276(42.2)	322(49.2)	9.30	9.5 × 10^−3^	**0.029**
	PM	200(27.5)	526(72.5)	1.13(0.94–1.36)	0.193	0.579	21(5.8)	158(43.5)	184(50.7)	5.32	0.070	0.210
	IIM	588(28.9)	1446(71.1)	1.21(1.06–1.38)	4.3 × 10^−3^	**0.013**	77(7.6)	434(42.7)	506(49.7)	10.1	6.4 × 10^−3^	**0.019**
	Controls	644(25.2)	1916(74.8)				86(6.7)	472(36.9)	722(56.4)			
rs4937362		C	T				CC	TC	TT			
	DM	395(30.4)	905(69.6)	0.91(0.79–1.05)	0.190	0.570	63(9.7)	269(41.4)	318(48.9)	1.73	0.422	NS
	PM	208(28.8)	514(71.2)	0.84(0.70–1.00)	0.062	0.186	32(8.9)	144(39.9)	185(51.2)	3.36	0.187	0.561
	IIM	603(29.8)	1419(70.2)	0.79(0.68–0.91)	0.056	0.168	95(9.4)	413(40.9)	503(49.7)	3.60	0.165	0.495
	Controls	831(32.5)	1729(67.5)				145(11.3)	541(42.3)	594(46.4)			

Bold values are statistically significant (*P* < 0.05) (corrected for multiple comparisons by the Bonferroni adjustment test).

PM: polymyositis; DM: dermatomyositis; IIM: idiopathic inflammatory myopathies; OR: odds ratio; CI: confidence interval; χ^2^: Chi-square test; *Pc*: *P* value *c*orrected by Bonferroni method; NS: not significant.

**Table 3:** Analysis of the three SNPs based on three genetic models.

SNPs	Groups	Additive model		Dominant model		Recessive model	
		*Pc*	OR (95%CI)	*Pc*	OR (95%CI)	*Pc*	OR (95%CI)
rs6590330	PM	0.127	1.20(1.00–1.43)	**0.029**	1.37(1.08–1.74)	NS	1.03(0.71–1.50)
	DM	**0.030**	1.20(1.04–1.39)	**0.038**	1.28(1.05–1.55)	0.405	1.25(0.93–1.66)
	IIM	**0.010**	1.20(1.06–1.36)	**4.86** × **10** ^**−3**^	1.31(1.11–1.55)	0.704	1.17(0.90–1.51)
rs7117932	PM	0.577	1.13(0.94–1.36)	0.160	1.26(1.00–1.59)	NS	0.85(0.52–1.39)
	DM	**8.79** × **10** ^**−3**^	1.25(1.08–1.45)	**8.37** × **10** ^**−3**^	1.33(1.10–1.61)	0.427	1.30(0.92–1.85)
	IIM	**0.013**	1.21(1.06–1.38)	**4.54** × **10** ^**−3**^	1.31(1.11–1.54)	NS	1.13(0.83–1.57)
rs4937362	PM	0.202	0.85(0.71–1.01)	0.312	0.82(0.65–1.04)	0.551	0.76(0.51–1.14)
	DM	0.593	0.91(0.79–1.05)	0.886	0.90(0.75–1.09)	0.821	0.84(0.61–1.15)
	IIM	0.179	0.89(0.78–1.00)	0.334	0.87(0.74–1.03)	0.403	0.81(0.62–1.07)

Bold values are statistically significant (*P* < 0.05) (corrected for multiple comparisons by the Bonferroni adjustment test).

PM: polymyositis; DM: dermatomyositis; IIM: idiopathic inflammatory myopathies; OR odds ratio; CI confidence interval; *Pc*: *P* value *c*orrected by Bonferroni method; NS: not significant.

### Association between *ETS1* polymorphisms and the ILD phenotype of IIM patients

We also investigated the potential associations of these SNPs in IIM patients with and without ILD. We compared ILD patients with negative patients and with all healthy controls. We also compared IIM cases without ILD with all healthy controls (Table 4). The associations of the rs7117932 and rs6590330 SNPs were statistically significant in DM and IIM patients complicated with ILD (all *P*_*c*_ < 0.05; Table 4). However, rs4937362 was not associated with PM or DM complicated with ILD in this cohort.

**Table 4:** Association between the SNPs and IIM with ILD.

Disease	Group	rs6590330		rs7117932		rs4937362	
		*Pc*	OR (95%CI)	*Pc*	OR (95%CI)	*Pc*	OR (95%CI)
DM	P vs. N	NS	1.10(0.87–1.38)	NS	1.11(0.87–1.41)	NS	1.10(0.87–1.40)
	P vs. C	**0.031**	1.24(1.05–1.47)	**8.90** × **10** ^**−3**^	1.31(1.10–1.56)	NS	0.94(0.79–1.12)
	N vs. C	0.63	1.13(0.93–1.38)	0.35	1.18(0.96–1.46)	0.41	0.86(0.70–1.05)
PM	P vs. N	NS	0.90(0.66–1.21)	NS	0.99(0.71–1.37)	0.91	0.85(0.61–1.17)
	P vs. C	0.82	1.13(0.91–1.41)	NS	1.13(0.89–1.43)	0.16	0.78(0.61–0.99)
	N vs. C	0.16	1.26(1.00–1.59)	0.95	1.14(0.88–1.47)	NS	0.92(0.72–1.18)
IIM	P vs. N	NS	1.02(0.85–1.22)	NS	1.07(0.88–1.30)	NS	1.00(0.83–1.22)
	P vs. C	**0.034**	1.20(1.04–1.39)	**0.016**	1.24(1.07–1.45)	0.35	0.89(0.76–1.03)
	N vs. C	0.13	1.18(1.00–1.39)	0.26	1.16(0.98–1.38)	0.41	0.88(0.74–1.04)

Bold values are statistically significant (*P* < 0.05) (corrected for multiple comparisons by the Bonferroni adjustment test). DM: dematomyositis; PM: polymyositis; IIM: idiopathic inflammatory myopathies; ILD: interstitial lung disease; Group P: patients with ILD; Group N: patients without ILD; Group C: Healthy controls; *Pc*: *P* value corrected by Bonferroni method. Group P (DM: n = 390; PM: n = 195; IIM: n = 585); Group N (DM: n = 264; PM: n = 168; IIM: n = 432); Group C (n = 1280).

## Discussion

In this study, we examined the association between *ETS1* polymorphisms and IIM patients in a northern Chinese Han population. Our study confirmed that two SNPs (rs7117932 and rs6590330) in the *ETS1* gene region are associated with IIM patients with and without ILD. It is interesting to note that stratification into traditional IIM clinical subgroups revealed an association with DM patients but not PM patients. Our experimental results suggest that the *ETS1* gene plays different roles in the pathogenesis of various clinical subgroups and will help improve our understanding of the different types of clinical interventions for these rare diseases.

Our experimental results were inconsistent for PM and DM, and several reasons may explain these findings. First, the immunopathology differs for PM and DM. Muscle biopsy of PM patients reveals that the primary inflammatory cells are lymphocytes (CD8-positive cells) that have invaded histologically healthy muscle fibers expressing MHC class I antigens^24–26^. Conversely, DM patients show a distinct immunohistopathologic phenotype, and muscle biopsy demonstrates a mononuclear inflammatory cell exudate that is predominantly perivascular or is located in the interfascicular septae rather than within the fascicles^24,25^. Second, the clinical characteristics of PM and DM are distinct. PM is characterized by symmetric proximal muscle weakness, and DM is characterized by an erythematous rash as well as symmetric proximal muscle weakness. Our data suggest that the roles of the *ETS1* gene in the pathogenesis of DM and PM are distinct, which is likely related to the unique features of PM and DM.

The *ETS1* gene encodes a member of the ETS family of transcription factors; these transcription factors are defined by the presence of a conserved ETS DNA-binding domain that recognizes the core consensus DNA sequence GGAA/T in target genes. Ets1 is expressed at high levels mainly in immune tissues such as the thymus, spleen, and lymph nodes (B cells, T cells, natural killer (NK) cells, NK T cells and non-lymphoid immune cells). The enforced expression of Ets1 blocks the differentiation of B and T cells. Knockdown of Ets1 causes multiple defects in the immune system. Ets1 plays numerous roles, including regulating the development of T-cell subsets, binding to and trans-activating cytokine and chemokine genes, and controlling the expression or activity of downstream effectors of the cytokine signaling pathways. John *et al*.^27^ reported that Ets1 regulates the differentiation of plasma cells by inhibiting the activity of Blimp-1. Conversely, Ets1 can promote the differentiation of T1 cells and restrain the differentiation of Th17 cells^28,29^, Ets1 can also affect the development of NK cells and NK T cells^30^. A previous study confirmed that *ETS1* plays a role in immune regulation and the occurrence of SLE^31^. We found that two *ETS1* SNPs (rs7117932 and rs6590330) were associated with the development of IIM alone or IIM with ILD in a northern Chinese Han population. We also found that IIM patients might share a gene commonly associated with the risk of other autoimmune diseases. However, the effects of these two *ETS1* SNPs (rs7117932 and rs6590330) on the function of Ets1 are unknown, and the specific mechanism of *ETS1* gene in the development of IIMs is unclear. Therefore, functional studies of *ETS1* gene and other gene on the pathogenesis of IIMs are imminent.

IIMs are a heterogeneous group of rare autoimmune diseases, and genetic studies on these diseases are limited. Additionally, the majority of early candidate gene association studies have only included small sample populations. Our study was the first comprehensive investigation to examine the relationship between the *ETS1* gene and IIM patients from a northern Chinese Han population. The clinical features of these patients were consistent with the international guidelines^21,22^. Our sample size had the power (greater than 85%) to detect moderate or even marginal effects. However, our study still has limitations associated with explaining the experimental observations. For example, we did not examine the function of *ETS1* variants in the development of IIM or analyze the potential association of these genetic variants with the serological phenotypes (autoantibody profiles) of IIM patients.

In summary, this was the first comprehensive genetic study to examine the association between common variants in the *ETS1* gene and IIM patients in a northern Chinese Han population. The results indicate that *ETS1* gene polymorphisms (rs7117932 and rs6590330) are associated with IIM alone and IIM with ILD in a northern Han Chinese population.
